# Axonal degeneration in the anterior insular cortex is associated with Alzheimer’s co-pathology in Parkinson’s disease and dementia with Lewy bodies

**DOI:** 10.1186/s40035-022-00325-x

**Published:** 2022-12-07

**Authors:** Yasmine Y. Fathy, Laura E. Jonkman, John J. Bol, Evelien Timmermans, Allert J. Jonker, Annemieke J. M. Rozemuller, Wilma D. J. van de Berg

**Affiliations:** 1 Amsterdam UMC, Department of Anatomy and Neurosciences, Section Clinical Neuroanatomy and Biobanking, Amsterdam Neuroscience, Vrije University Amsterdam, O|2 Life Sciences building, De Boelelaan 1108, 1081 HZ Amsterdam, Netherlands; 2 Amsterdam Neuroscience, Program Neurodegeneration, Amsterdam, the Netherlands; 3 Amsterdam UMC, Department of Pathology, Amsterdam Neuroscience, Vrije University Amsterdam, De Boelelaan, Amsterdam, Netherlands; 4 Department of Neurology, Erasmus Medical Center, Postbus 2040, 3000 CA Rotterdam, Netherlands

**Keywords:** α-Synuclein, Insular subregions, Axonal length density, Alzheimer’s disease pathology, Neurofilament, Myelin

## Abstract

**Background:**

Axons, crucial for impulse transmission and cellular trafficking, are thought to be primary targets of neurodegeneration in Parkinson’s disease (PD) and dementia with Lewy bodies (DLB). Axonal degeneration occurs early, preceeding and exceeding neuronal loss, and contributes to the spread of pathology, yet is poorly described outside the nigrostriatal circuitry. The insula, a cortical brain hub, was recently discovered to be highly vulnerable to pathology and plays a role in cognitive deficits in PD and DLB. The aim of this study was to evaluate morphological features as well as burden of proteinopathy and axonal degeneration in the anterior insular sub-regions in PD, PD with dementia (PDD), and DLB.

**Methods:**

α-Synuclein, phosphorylated (p-)tau, and amyloid-β pathology load were evaluated in the anterior insular (agranular and dysgranular) subregions of post-mortem human brains (*n* = 27). Axonal loss was evaluated using modified Bielschowsky silver staining and quantified using stereology. Cytoskeletal damage was comprehensively studied using immunofluorescent multi-labelling and 3D confocal laser-scanning microscopy.

**Results:**

Compared to PD and PDD, DLB showed significantly higher α-synuclein and p-tau pathology load, argyrophilic grains, and  more severe axonal loss, particularly in the anterior agranular insula. Alternatively, the dysgranular insula showed a significantly higher load of amyloid-β pathology and its axonal density correlated with cognitive performance. p-Tau contributed most to axonal loss in the DLB group, was highest in the anterior agranular insula and significantly correlated with CDR global scores for dementia. Neurofilament and myelin showed degenerative changes including swellings, demyelination, and detachment of the axon-myelin unit.

**Conclusions:**

Our results highlight the selective vulnerability of the anterior insular sub-regions to various converging pathologies, leading to impaired axonal integrity in PD, PDD and DLB, disrupting their functional properties and potentially contributing to cognitive, emotional, and autonomic deficits.

## Introduction

The presence of α-synuclein pathology and loss of dopaminergic neurons are known as the main neuropathological hallmarks of Parkinson’s disease (PD) and dementia with Lewy bodies (DLB). PD and DLB are two of the most common and progressive neurodegenerative diseases affecting more than 6 million people worldwide, and both lack disease-modifying treatments [1, 2]. Post-mortem human studies have shown that during the first 3 years after PD diagnosis, approximately 35%–75% of striatal nerve terminals are already lost compared to the loss of dopaminergic neurons which lags behind [3, 4]. The magnitude of nerve terminal loss compared to that of neurons indicates that axons could be primary targets of degeneration while neurons die as a result of a “dying back” process [5, 6]. Axonal loss has generally been found to occur quite early [3, 4, 6–8], precedes neuronal loss [5, 9], and affects both the central and peripheral nervous systems [10, 11]. Pradoxically, axons are also thought to contribute to the spread of pathological aggregates through α-synuclein transport across synaptically connected regions [12]. Not all axons are equally vulnerable to degeneration; long, thin, densely arborized, and poorly myelinated axons of projection neurons are found most susceptible to Lewy pathology and degeneration [13–15]. These axons generally have high metabolic demands as well as proteostatic burden [16]. Overall, axonal damage and loss of connectivity are increasingly associated with cognitive dysfunction, thus representing a potentially relevant biomarker for cognitive impairment in PD, a burdening multi-faceted aspect of the disease [17, 18].

According to our knowledge, the morphological features of axonal degeneration and myelin change in PD and DLB have not yet been well described outside the nigrostriatal circuitry. We recently discovered that the insular cortex, a brain hub with crucial integrative functions, is highly vulnerable to α-synuclein pathology in PD and DLB [19]. We observed a severe load of Lewy pathology and astroglial degenerative changes in the anterior insula most prominent in PD with dementia (PDD) and DLB donors. The insular cortex is broadly connected to the whole brain and plays a role in affective, autonomic, somatosensory, and cognitive functions [20, 21]. The multi-functional capabilities of the insular cortex are driven by an underlying heterogeneous cyto-architecture, namely, anterior (agranular and dysgranular) and posterior granular, each with its own set of preferential connections. The insular cortex is thought to widely contribute to non-motor deficits in PD and shows early changes in PD with mild cognitive impairment as well as in prodromal PD and DLB [22–24].

In the current study, we comprehensively evaluated the morphological features of axonal and cytoskeletal degeneration, concomitant pathology, and their relationship with axonal loss in the anterior insular cortex. Particularly in DLB, Alzheimer’s disease (AD)-related pathology is commonly present and contributes to a specific clinical phenotype showing more rapid cognitive decline and shorter survival time. In fact, 18% of DLB patients have advanced AD-related neuropathology and 28% have sufficient post-mortem AD neuropathology to receive a secondary diagnosis of AD [25]. We hypothesize that axonal degeneration is most severe in the agranular compared to the dysgranular insula in PD(D) and DLB and associated with the local burden of α-synuclein, phosphorylated tau (p-tau), and amyloid-β pathology. In a well-characterized cohort of PD(D) and DLB brain donors (*n* = 27), we studied axonal degeneration using modified Bielschowsky silver staining, multicolour immunofluorescence combined with advanced confocal laser-scanning microscopy (CSLM), and stereological principles. Using 3D microscopy, we provide an overview of morphological features of proteinopathy as well as axonal and cytoskeletal degeneration in the anterior insular subregions of PD(D) and DLB donors.

## Materials and methods

### Post-mortem brain tissues

Formalin-fixed postmortem human brain tissues from 27 donors with PD, PDD, and DLB (range = 60–93 years) were collected in close collaboration with the Netherlands Brain Bank (NBB, Amsterdam, The Netherlands; www.brainbank.nl). In compliance with ethical and legal guidelines, all donors or next-of-kin had provided written informed consents for donation of brain tissue and access to their clinical and neuropathological reports for research purposes. The brain donor program of the NBB was approved by the local medical ethics committee of the VU University Medical Center, Amsterdam. The main inclusion criteria for the current study were: (i) clinical diagnosis of PD, PDD, or DLB according to revised MDS and McKeith diagnostic criteria [26, 27] and (ii) neuropathological confirmation of diagnosis [28]. Retrospective chart reviews were carried out for all included donors and the latest clinical dementia rating (CDR) global scores were retrieved. The CDR global score is a scoring algorithm designed to assess the overall severity of dementia. It is based on cognitive and functional deficits in the domains of memory, orientation, judgement, problem-solving, hobbies, personal care, and community affairs. Ratings are assigned on a 5-point scale ranging from 0 (normal) to 3 (severe) [29]. Subjects were excluded if they had a long history of neuropsychiatric disorders or concomitant neurological disorders, infarcts and other abnormalities within the insular cortex (Table 1).

**Table 1:** Subject demographics and neuropathological staging

ID & clinical diagnosis	Sex	Age death (years)	Disease duration (years)	CDR	Braak *α*-synuclein	Braak NFT & age-related tauopathy	Thal phase	CAA	PMD (h:min)	ABC score
**PD_1**	M	77	11	-	5	II	1	–	5:35	A1B1C0
**PD_2**	F	68	16	0.5	5	II	1	–	7:55	A1B1C0
**PD_3**	F	73	3	0	4	II, ARTAG	2	–	6:10	A1B1C0
**PD_4**	M	91	26	0.5	6	III, PART	3	Type I	4:00	A3B2C1
**PD_5**	M	57	16	–	5	**0**	1	-	06:35	A1B0C0
**PDD_1**	F	88	15	0.5	5	II, ARTAG and PART	0	–	05:35	A0B1C0
**PDD_2**	M	74	7	1	6	II, ARTAG	3	–	04:35	A2B1C0
**PDD_3**	F	74	13	1	6	III	3	Type II, grade 1	03:45	A2B2C1
**PDD_4**	F	81	8	0.5	6	II, ARTAG	3	Type I, grade 1	06:40	A2B1C1
**PDD_5**	M	75	6	0.5	6	II, ARTAG	1	–	03:35	A1B1C0
**PDD_6**	M	70	19	1	6	III, ARTAG	3	–	04:30	A2B2C0
**PDD_7**	F	88	6	0.5	6	II, ARTAG	1	–	06:05	A1B1C0
**PDD_8**	M	81	18	0.5	6	III, ARTAG & PART	0	–	06:10	A0B2C0
**PDD_9**	F	83	14	-	6	IV, ARTAG & PART	0	-	06:15	A0B2C0
**PDD_10**	M	71	26	3	5	II, ARTAG	1	–	13:50	A1B1C0
**PDD_11**	F	87	10	2	5	III, ARTAG	3	–	07:55	A2B2C0
**PDD_12**	M	94	18	3	6	III, ARTAG	2	Type II, stage 1	06:30	A1B2C1
**PDD_13**	M	75	12	1	5	II, ARTAG	0	–	12:25	A0B1C0
**DLB_1**	M	67	3	3	6	V, ARTAG	5	Type I	05:20	A3B3C3
**DLB_2**	M	75	2	3	6	III, ARTAG	4	–	05:00	A2B2C3
**DLB_3**	M	81	4	2	6	III	3	–	08:21	A2B2C0
**DLB_4**	M	78	6	1	6	I, ARTAG	3	Type II	05:10	A2B1C0
**DLB_5**	M	60	7	3	6	0	0	-	08:30	A0B0C0
**DLB_6**	M	70	5	-	6	III, ARTAG	4	Type I, grade 1	07:40	A3B2C1
**DLB_7**	M	71	9	–	6	II, ARTAG	3	Type I, grade 1	04:10	A2B1C1
**DLB_8**	F	77	4	3	6	V, ARTAG	4	Type I, stage 1	03:45	A3B3C3
**DLB_9**	F	67	3	3	5	III	4	Type II, stage 1	07:45	A3B2C1

ABC score: A-C(0–3, *A*: Amyloid plaque score, *B*: Braak NFT score, *C*: CERAD neuritic plaque score), *ARTAG* Aging-related tau astrogliopathy, Braak NFT: 0-VI, Braak *α*-synuclein: 0–6, *CAA* Cerebral amyloid angiopathy (types: I (capillary) or II (capillary and arteriolar), stages 1–3), *DLB* Dementia with Lewy bodies, *NFT* Neurofibrillary tangles, *PART* Primary age-related tauopathy, *PD* Parkinson’s disease, *PDD* PD dementia, *PMD* Post-mortem delay, Thal phase: 0–5

Brain dissection was performed according to international guidelines of Brain Net Europe II (BNE) consortium (http://www.brainnet-europe.org) and NIA-AA by an experienced neuropathologist (NBB: AMR). The entire insula was dissected into 0.5–1-cm thick blocks according to its anatomical borders with surrounding brain regions. Using the central sulcus as a landmark separating the anterior from the posterior insula, the anterior insular cortex (AIC) was then dissected further [30]. Tissue blocks were cryo‐protected with 30% sucrose, frozen and sectioned using a sliding microtome into 60-μm thick sections and these were stored at − 30 °C until further processing. Sections were first incubated in various gradients of alcohol followed by xylene to induce de-fatting, and then stained with Nissl. This allowed the identification of the agranular and dysgranular sub-regions based on the absence or presence of layers II and IV by two experienced researchers (YF and LJ) as previously described [19, 31].

### Neuropathological assessment

For neuropathological diagnosis and staging, 6-μm paraffin sections from brainstem, limbic and neocortical brain tissue blocks of all donors were immunostained for α‐synuclein (clone KM51, 1:500, Monosan Xtra, The Netherlands), amyloid-β (clone 6F/3D, 1:500, Dako, Denmark), phosphorylated tau (p-tau, clone AT8, 1:500, Thermo Fisher Scientific, Rockford, IL), haematoxylin and eosin (H&E), TDP‐43 and congo red according to current diagnostic guidelines of BrainNet Europe [28]. Pathological staging protocols were based on Braak α‐synuclein (Braak α‐syn 0–6), Braak staging for neurofibrillary tangles (Braak NFT 0–6), Thal phase for amyloid-β (0–5), and CERAD score for neuritic plaques [32–36]. Glial tauopathy such as age-related tauopathy of the astroglia (ARTAG) and primary age-related tauopathy were assessed primarily in the temporal cortex, olfactory cortex and amygdala by an experienced neuropathologist (AMR) [37, 38]. Clinical symptoms and diagnosis ante-mortem, along with neuropathological scores, were used to provide a final pathological diagnosis. DLB diagnosis was made based on the revised criteria for the clinical diagnosis of probable and possible DLB using patient’s ante-mortem clinical history, neurological, and psychiatric assessments [26]. Furthermore, using neuropathological assessment, DLB diagnosis was confirmed when there was concomitant AD pathology, cerebral amyloid angiopathy (CAA), and cortical Lewy pathology [39](Table 1).

### Evaluation of axonal degeneration using modified Bielschowsky silver staining

Modified Bielschowsky silver staining [40] was used for the evaluation of axonal degeneration in the anterior insula. Prior to staining, all glassware was thoroughly rinsed with purified water for several hours or overnight and metals were avoided. All incubations were performed at cold temperature (5 °C) [40]. Free-floating 60-μm tissue sections (interval: 1 in 20) were rinsed in distilled water followed by Tris-buffered saline (TBS, pH 7.6) and incubated in 20% silver nitrate solution (AgNO_3_; Sigma, CAS No: 7761-88-8) for 30 min at 5 °C in the dark. Subsequently, non-evaporated ammonium hydroxide 30%–33% (NH_3_) solution was slowly added to the sections until the color turned black, and then incubated for 30 min at 5 °C in the dark (Honeywell, CAS No: 1336-21-6). Sections were then rinsed with evaporated ammonia-water for 10 min (100 µl ammonia in 50 ml distilled water). Subsequently, a developer solution was freshly prepared, which consisted of 100 ml distilled water, 20 ml 37% formalin (Sigma, CAS No: 50–00-0), 1 drop nitric acid (Fisher, CAS No: 7697-37-2), and 0.5 g citric acid (Sigma, CAS No: 77-92-9). Sections were incubated in the developer solution at 5 °C in the dark for visualization of the reduced metallic silver. Staining was monitored carefully and the incubation was stopped within 10–15 min. This was followed by rinsing in Hypo (sodium thiosulfate 5% in distilled water; Honeywell, CAS No: 7772-98-7) to stabilize the developing reaction and remove free AgNO_3_ deposits from the tissue. Sections were then rinsed, mounted with 0.3% gelatin (Oxoid), dried and cover-slipped using Entellan (Sigma).

### Evaluation of α‐synuclein, p-tau, and amyloid-β pathology in the anterior insular subregions

For α‐synuclein immunostaining, consecutive free‐floating 60-μm thick sections of the anterior insula were pre-treated with 98% formic acid (Sigma‐Aldrich, Darmstadt, Germany) and incubated with primary antibody mouse anti‐α‐synuclein (syn-1, 1:2000, 610786, BD Biosciences, Berkshire, UK) in TBS and 0.5% TritonX-100 solution overnight at room temperature, as previously described by Braak and colleagues [41]. For immunostaining with antibodies against p-tau and amyloid-β, free-floating sections were rinsed with TBS and pre-treated with citrate buffer (pH 6.0) in a steamer (95 °C) for antigen-retrieval, followed by 80% formic acid for amyloid-β. Non-specific staining was then blocked with 0.3% H_2_O_2_ and 0.1% sodium azide in TBS followed by 2% normal goat serum. Subsequently, sections were incubated in primary antibody mouse anti-p-tau (1:1000, MN1020, AT8, Thermofisher-scientific, Netherlands) and adjacent sections were incubated in mouse anti-amyloid-β (1:1000, M08720, clone 6f/3d, Dako, Denmark) diluted in TBS and 0.1% Triton-X overnight at 4 °C. All sections were incubated with biotinylated secondary antibody IgG (1:200, Vector Laboratories, Burlingame, CA) followed by standard avidin‐biotin complex (1:200, Vectastatin ABC kit, Standard; Vector Laboratories) in TBS for 2 h. Tissue samples were incubated in 3,3′‐diaminobenzidine to visualize staining and were mounted and counter‐stained with thionin (0.13%, Sigma‐Aldrich, Darmstadt, Germany). Immunofluorescent double staining of α-synuclein and glial fibrillary acid protein (GFAP) was performed as described elsewhere [19].

Load of α-synuclein, p-tau and amyloid-β pathology was calculated as percentage immunoreactivity per surface area in each region of interest (ROI) using area fraction plugin in ImageJ (1.52n) [42]. Anterior insular sub-regions (agranular and dysgranular) were identified on adjacent Nissl-stained sections, and the same borders were used to define ROIs on sections immuno-stained for pathological aggregates [19].

### Multi-labelling immunofluorescence for evaluation of axonal cytoskeletal abnormalities

We included multi-labelling immunofluoresecent staining with axonal and myelin markers to visualize the axonal morphology and cytoskeletal abnormalities in 3D with CSLM. Adjacent 60-μm thick sections from PD(D) and DLB cases were rinsed in TBS, pre-treated with EDTA-Tris (pH 9.0) in a steamer (95 °C) and incubated in a cocktail of the following primary antibodies: (1) mouse  anti-myelin proteolipid protein (PLP, 1:500, MCA839G, plpc1, Bio-Rad, Netherlands) and (2) chicken anti-neurofilament heavy chain (NfH, 1:500, AB5539, heavy-chain, Millipore) diluted in TBS, 2% normal donkey serum, and 0.5% Triton-X. Immunostaining was performed for 48 h at 4 °C followed by incubation with secondary antibody goat anti‐chicken coupled with Alexa Fluor 594 (1:400; Molecular Probes, Waltham, MA), donkey anti‐mouse coupled with Alexa Fluor 647, and diamidino‐2‐phenylindole [4, 6] dihydrochloride (DAPI; Sigma) for nuclear staining for 2 h in the dark. Tissue samples were subsequently rinsed in TBS and blocked in 5% normal mouse serum for 1 h followed by incubation with Alexa488-conjugated mouse anti-phosphorylated-Serine129 (pSer129) α‐synuclein antibody (1:100, 11A5, gift from Prothena Biosciences Inc., San Francisco, CA) for 2 h at 4 °C in the dark. The tissue sections were then mounted on glass slides and cover‐slipped with mowiol-DABCO as a mounting medium (4‐88 Calbiochem).

### Microscopic imaging

Digital images of the immunostained slides were made with a photomicroscope (Leica DM5000) equipped with a color camera (DFC450), Leica LASV4.4 software and 63 × oil objective lens. Immunofluorescent labelling was visualized using CLSM LEICA TCS SP8 (Leica Microsystems, Jena, Germany). Adjacent Nissl-stained sections were used for delineation of subregions and the ROIs were superimposed on the images of the immunofluorescent stained sections. This was followed by sampling of all axons (Bielschowsky staining) in the superficial and deep layers of each sub-region. Image acquisition was done using 100 × /1.4 NA objective lens, 405 nm diode, and pulsed white light laser (80 Hz) with excitation wavelengths of 405, 499, 598, and 653 nm, scanned using frame/stack sequential mode. For optimal resolution, z-step size was calculated for each scan based on the Nyquist-Shannon sampling theorem [43] and line accumulation/averaging were used as deemed appropriate per channel. Deconvolution of image stacks was performed using Huygens Professional software (Scientific Volume Imaging, Hilversum, the Netherlands). Images are shown as maximum projections of all channels combined and all figures were composed using Adobe Photoshop (Adobe Systems Incorporated). Colocalization between GFAP and α-synuclein was assessed using Imaris 8.3 (Bitplane, South Windsor, CT).

### Stereological analysis of axonal loss

Axonal length density, based on Bielschowsky silver impregnated sections, was quantified using the stereological space balls probe from stereoinvestigator software (MBF Bioscience, Williston, VT) and Leica microscope DMR HC (v2019.01.4) [44–46]. Serial sections (1:20, Range of sections: 3–8) were used for quantification, and counting parameters were chosen to allow counting ≥ 200 axon intersections for each sub-region. A sphere (radius = 10 μm) was used along with a sampling grid 2700 μm × 2700 μm. ROIs corresponding to the grey matter of the agranular and dysgranular sub-regions were drawn at a low magnification (using a 2.5 × objective) and axonal quantification was completed at higher magnification (100 × oil objective). Only nerve fibers were counted and when a counting frame/sphere contained tangles or other pathologies, they were not counted and the following counting frame was used. Moreover, a fiber was counted only when it fully intersected with the sphere at least once. Tissue thickness was measured manually at each sampling frame (mean, 22 ± 1.42 μm) and coefficient of error (CE) was calculated for each sub-region (mean CE agranular = 0.13 ± 0.06; CE dysgranular = 0.12 ± 0.05).

To calculate estimated axonal length (L), total number of intersections of fibers with space balls (Qi) throughout all sections was multiplied by the volume of sampling frame. The volume (volume = grid X × grid Y × section thickness) is divided by the surface area of sphere (a = $4\times \pi \times {r}^{2}$) multiplied by the reciprocal of sampling fraction of the section (1:20) [44, 46]. To calculate density, total axonal length (L) was divided by sampled reference volume per ROI. Reference volume was derived through planimetry, calculated as a measure of total area of the ROI multiplied by section height [44, 46].

### Statistical analysis

Statistical analyses were performed using SPSS (version 26.0, IBM) and graphs were made using graph prism, version 9. When tissue or staining quality was insufficient, cases were omitted from analysis. Demographics of controls, PD, PDD and DLB were compared using chi-square test for categorical data and ANOVA, with age corrections, for numerical data. To analyze differences in axonal length density between insular sub-regions (agranular and dysgranular) across all three patient groups (PD, PDD, DLB), an ANCOVA, with adjustment for age, and *post-hoc* paired *t*-test were used. Quantitative scores for p-tau and α-synuclein pathology load showed non-normal distribution and were log-transformed followed by parametric analysis with ANCOVA using age as a covariate followed by *post-hoc* paired *t*-test for group differences. For amyloid-β, due to the presence of multiple zero scores from pathology quantification, scores were dichotomized (0 or 1) and analyzed using logistic regression. To assess the pathology and group effects on axonal density, variables were pooled into the model and a nested linear mixed model analysis was performed using backward elimination. Axonal length density was the dependent variable, area% load of p-tau, α-synuclein, and amyloid-β were main effects and age was a covariate. Spearman’s rank correlation was computed to assess the relationship between CDR scores and age, group, disease duration, axonal density, p-tau, α-synuclein, and amyloid-β scores. Analyses were then re-computed per gender. Correction for multiple comparisons was not performed. Statistical significance was set at *P* < 0.05.

## Results

### Cohort description

All included donors had moderate to severe disability (estimated Hoehn and Yahr scale, 4–5). DLB donors were mostly males (DLB = 78% vs PDD = 54% males), significantly younger than PDD donors (71 ± 6.1 years vs 81.4 ± 8.4 years; *P* = 0.02) and had a significantly shorter disease duration (5 ± 2.4 years) compared to both PD and PDD (18 ± 4 years and 13.4 ± 5 years, respectively; *P* = 0.001). All disease groups had advanced Braak α-synuclein stages, whereas Braak NFT stages were most advanced in PDD and DLB, and accompanied by glial tauopathy (ARTAG). The DLB group also had advanced amyloid-β Thal phases (range: 3–5) except for one younger patient (DLB_5, Thal phase = 0). CAA was more frequent (66% of cases) in the DLB group compared to PDD (25%) and PD (20%) and included both type I and type II CAA. The demographics, age-of-onset, disease duration, CDR scores, and neuropathological staging of all donors included in this study are summarized in Table 1.

### Clinico-pathological correlates in PD, PDD and DLB

CDR global scores (*n* = 22) showed a significant positive correlation with group as well as p-tau scores, indicating that worse cognitive performance (higher scores) correlates with DLB group (*r*(42) = 0.73; *P* < 0.001) and higher p-tau load (*r*(38) = 0.37, *P* = 0.017). We also found a significant negative correlation between CDR scores and disease duration, indicating a shorter disease duration correlates with worse cognitive performance (*r*(42) =  − 0.4; *P* = 0.008). A significant negative correlation was also found between CDR scores and axonal length density within the dysgranular insula (r(17) =  − 0.5; *P* = 0.04). Correlations computed based on gender showed significant results only for the female gender, including a significant negative correlation between CDR scores and disease duration (r(16) =  − 0.74; *P* < 0.001) as well as axonal density (*r*(16) =  − 0.52, *P* = 0.02*)*, and a significant positive correlation between CDR scores and p-tau (*r*(15) = 0.82, *P* < 0.001) as well as α-synuclein scores (*r*(10) = 0.93; *P* < 0.001).

### Morphology of α-synuclein, p-tau, and amyloid-β pathology

**Fig. 1:**
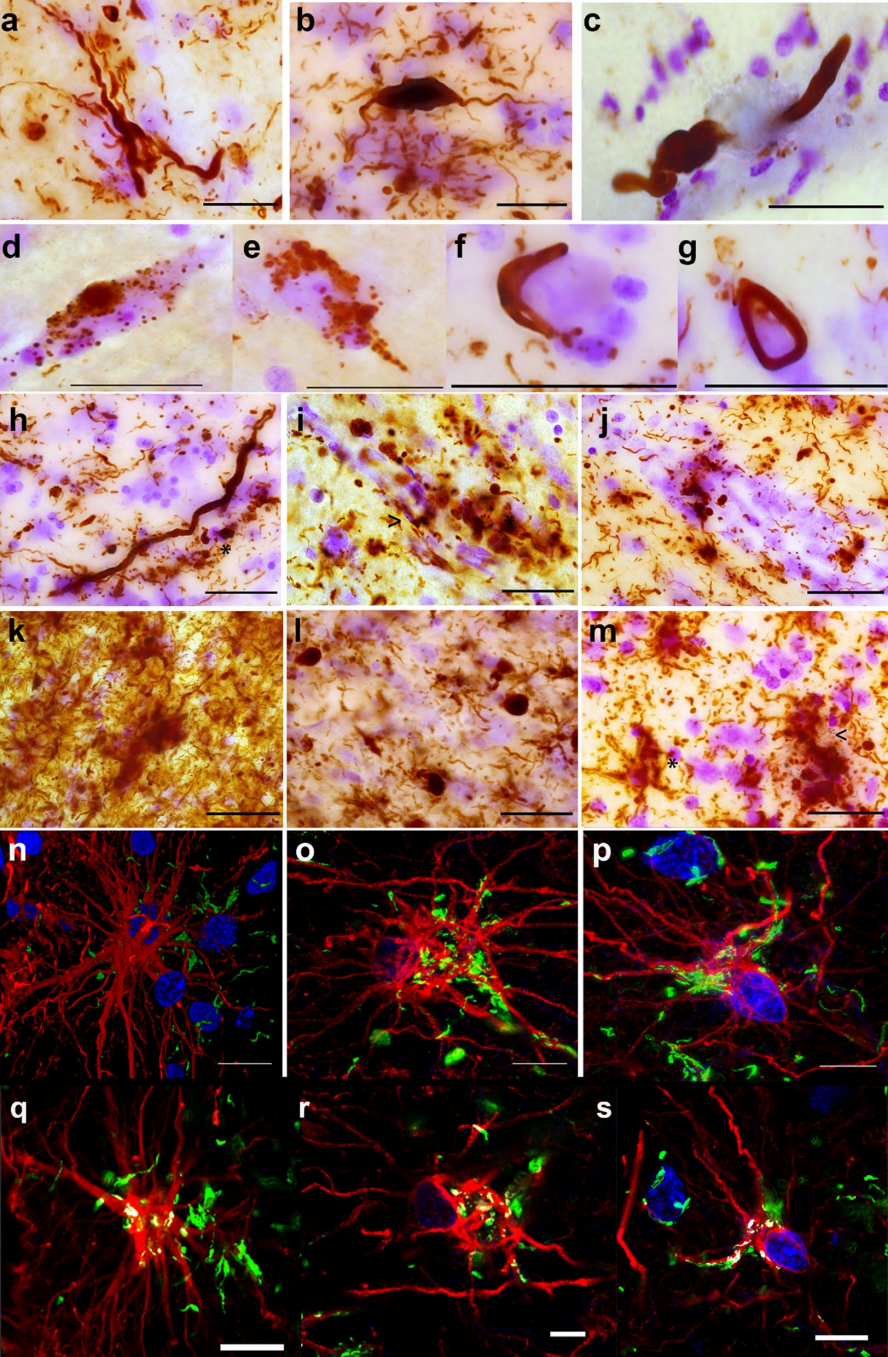
Morphological features of α-synuclein pathology (syn-1) in the anterior insula of PD, PDD and DLB patients. In the agranular insula, LNs in PD_4 (**a**), a bulgy LN in PDD_1 (**b**), and LN in layer I in DLB_2 (**c**). Various LBs were observed in layers 5–6 and intracytoplasmic granular deposits in PD_5 and PD_3 (**d,e**), an α-synuclein double-string related to a cell, possibly a glial cell, and surrounding a neuronal soma in PDD_1 (**f**), and circular deposit in DLB_3 (**g**). Glial synucleinopathy and a long LN (*)  were seen in PDD_1 in deeper layers of agranular insula (**h**). Vascular synucleinopathy ( *) and glial synucleinopathy were seen in PDD_3 (**i,j**). In DLB subjects, dense α-synuclein deposits were seen in agranular insula (DLB_8, **k**), LNs and LBs in the agranular insula in DLB_1 and in the dysgranular insula (**l**), and star-shaped astrocytic synucleinopathy surrounded by iron deposits (*) in dysgranular insula in DLB_2 (**m**). Immunoflourescent staining of astroglial synucleinopathy (GFAP in red; *α*-synuclein in green) in the agranular insula in PD (**n**), in PDD_1 (**o**), and in DLB_2 (**p**) illustrate the extent of glial pathology in PD and DLB**. **Intensity-based colocalization analysis of GFAP and α-synuclein within same astrocytes shows colocalized voxels (yellow/white pixels; **q-s).** Magnification (630x), scale bars: 50 μm. DLB: dementia with Lewy bodies; GFAP: glial fibrillary acid protein; LBs: Lewy bodies; LNs: Lewy neurites; PD: Parkinson’s disease; PDD: Parkinson’s disease dementia

We observed Lewy bodies (LBs), mostly in pyramidal neurons of deeper layers (V-VI), and Lewy neurites (LNs) including bulgy LNs (Fig. 1a–c) across all layers in both subregions. Cytoplasmic granular α-synuclein deposits (Fig. 1d–e**),** string-shaped and circular α-synuclein deposits (Fig. 1f–g) as well as astrocytic synucleinopathy (Fig. 1h–j) were observed, particularly in the agranular insula of PD and PDD donors. The anterior insula in DLB donors showed more severe α-synuclein pathology with extensively dense LNs and astroglial star-shaped synucleinopathy (Fig. 1k–m). In one PDD donor, vascular synucleinopathy was observed in the anterior insula (Fig. 1i–j). Furthermore, by staining astrocytes, α-synucleinopathy was found within the GFAP-positive astrocyte cell bodies and surrounding processes in both sub-regions (Fig. 1n–p). Colocalization of GFAP and α-synuclein is also shown (Fig. 1q–s).

**Fig. 2:**
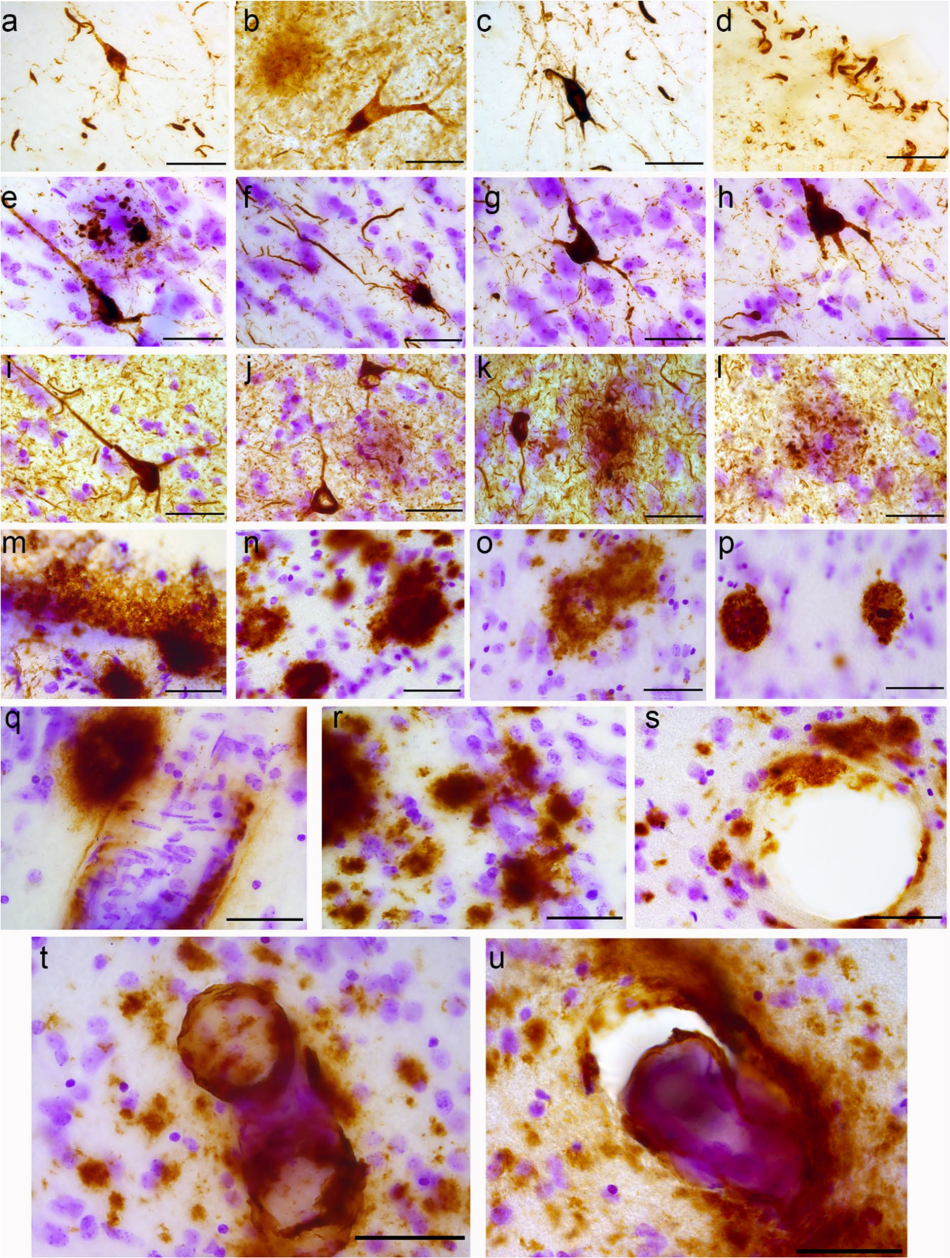
Alzheimer’s associated pathologies in the anterior insula of PD, PDD and DLB donors. In PD_3, a neurofibrillary tangle (NFT) is seen with distal swellings (**a**, **c**); in PD_4, p-tau immunoreactivity was seen in a fork cell (**b**) and subpial thorny astrocytes in the agranular insula are seen also in PD_3 (**d**). NFTs with fragmentation of distal fibers were observed in PDD_1 and PDD_4 (**e–h**). In DLB_2, more severe neuropil threads, NFT with fragmentation (**i**), ghost tangles (**j**) and neuritic plaque in DLB_1 (**k**), and glial tauopathy in DLB_2 (**l**) were seen. For amyloid-β (M08720, Dako), subpial pathology (**m**) and diffuse plaques (**n**) were seen in dysgranular insula in PD_4. In DLB_8, diffuse plaques with partial densification (**o**) and classic plaques (**p**) were seen. In PDD_3, CAA in dysgranular insula (**q**) and blood vessels surrounded by perivascular amyloid-β plaques (**r**) in agranular insula were seen. In DLB_8, peri-vascular and vascular amyloid-β pathology (**s**–**u**) were found surrounded by amyloid-β plaques. Magnification 630 × ; Scale bars: 50 μm. CAA: Cerebral amyloid angiopathy; DLB: dementia with Lewy bodies; NFT: neurofibrillary tangles; PD: Parkinson’s disease; PDD: Parkinson’s disease dementia

The anterior insula of the PD, PDD and DLB donors showed various degrees of severity for p-tau and amyloid-β pathology. NFTs, ghost tangles, glial tauopathy (including ARTAG and subpial thorny astrocytes), and neuritic plaques were observed in both sub-regions (Fig. 2a-l). We also observed fragmentation of NFTs in the agranular insula along with argyrophyllic grain disease (AGD) deposits in DLB (Fig. 2e-l). p-Tau pathology was also observed in a fork cell in deeper layers of the agranular insula (Fig. 2b). Neuropil threads, ghost tangles, and neuritic plaques were most prominent in DLB. Amyloid-β plaques were observed in the dysgranular insula and comprised of diffuse plaques as well as dense core plaques. Meningeal amyloid-β, CAA type I and II, and dyshorric amyloid (i.e. perivascular amyloid deposits) were most prominent in the dysgranular insula in DLB patients (Fig. 2m–u).

### Morphological features of axonal degeneration in the anterior insular sub-regions

**Fig. 3:**
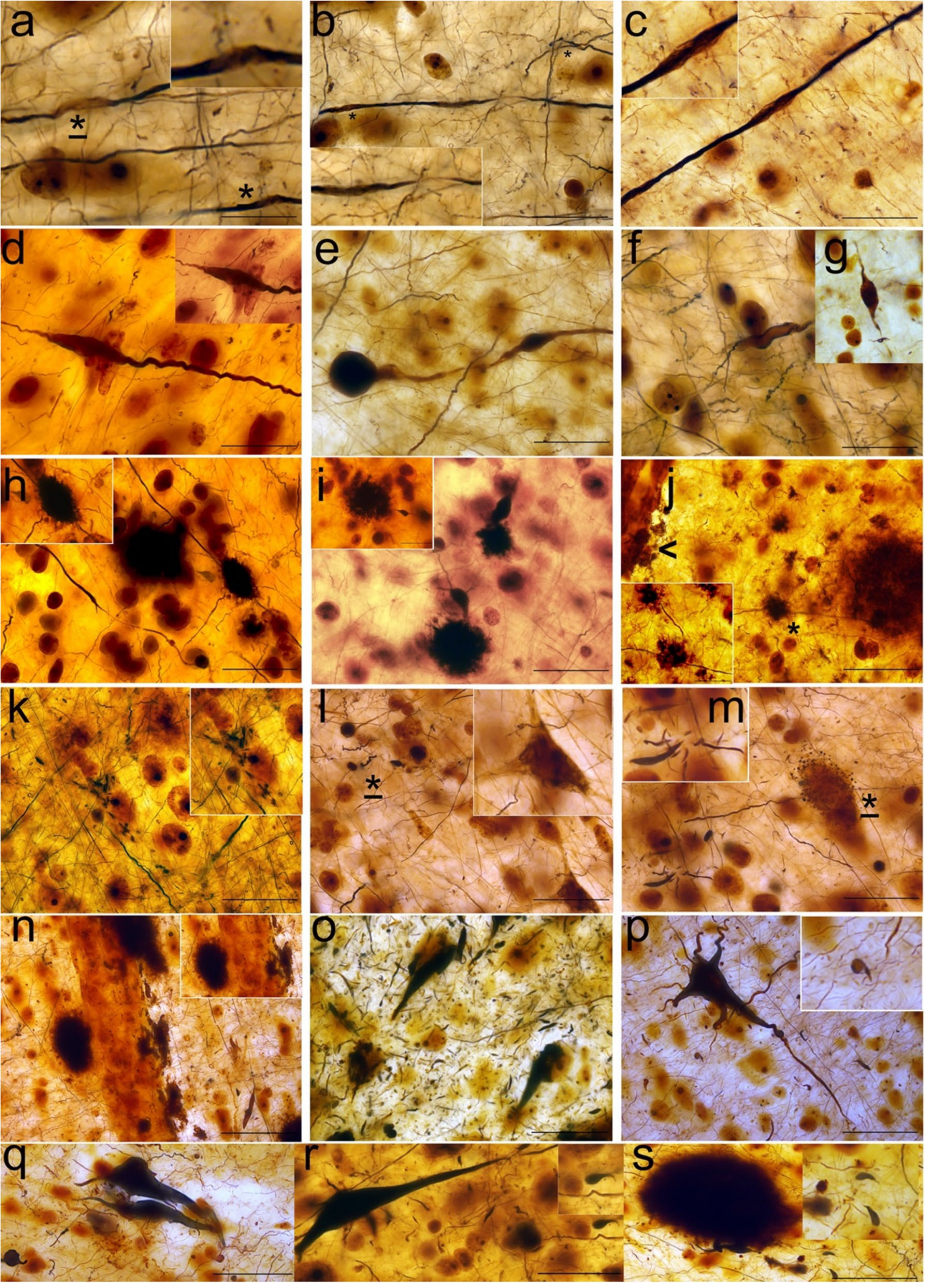
Morphological features of axonal degeneration and associated pathologies in the anterior insula visualized using Bielschowsky silver staining. Axonal thinning and associated myelin changes (*) were visible in fibers in PD_5 (**a**-**c**). Axonal thickening was seen in PD_4 dysgranular insula (**d**), thinning and spheroid body in PDD_1 agranular insula (**e**), and DLB_5 and DLB_7 in both sub-regions (**f, g**). Multiple fibers were surrounded by β-amyloid plaques in PD_3 (**j**) and PDD_3 with bulbous swelling in nearby axons in agranular insula (**h, i**). Early NFTs were seen in PD_3 dysgranular insula (**k**) and in PDD_6 agranular insula where dystrophic neurites and peri-somatic granules were also present (**l**, **m**). In DLB_9, multiple perivascular amyloid-β plaques were visible (**n**). NFTs as well as severe axonal degeneration were present in DLB_1 (**o**), DLB_8 (**r**) and DLB_9 which showed ghost tangles (**p**, **q**) surrounded by AG (**p**,**r**,**s**; upper right corner). AGs are featured as comma-shaped deposits. Magnification 630 × ; Scale bars: 50 μm. AG: argyrophilic grains; DLB: dementia with Lewy bodies; NFTs: neurofibrillary tangles; PD: Parkinson’s disease; PDD: Parkinson’s disease dementia

Neurodegenerative features such as localized axonal thickening, thinning, spheroids and myelin abnormalities were observed in the agranular insula in all donors (Fig. 3). In cases with more advanced Thal phases, amyloid-β plaques surrounding axons and bulbous axonal swellings were observed in the vicinity of the plaque (Fig. 3h–j). In PD donors, early NFT formation (pre-tangles) was seen associated with dystrophic neurites (Fig. 3k–m). NFTs maturing to ghost tangles were particularly present in DLB donors, where severe axonal degeneration and AGD deposits, seen as comma shaped deposits, were commonly seen (Fig. 3o–s).

**Fig. 4:**
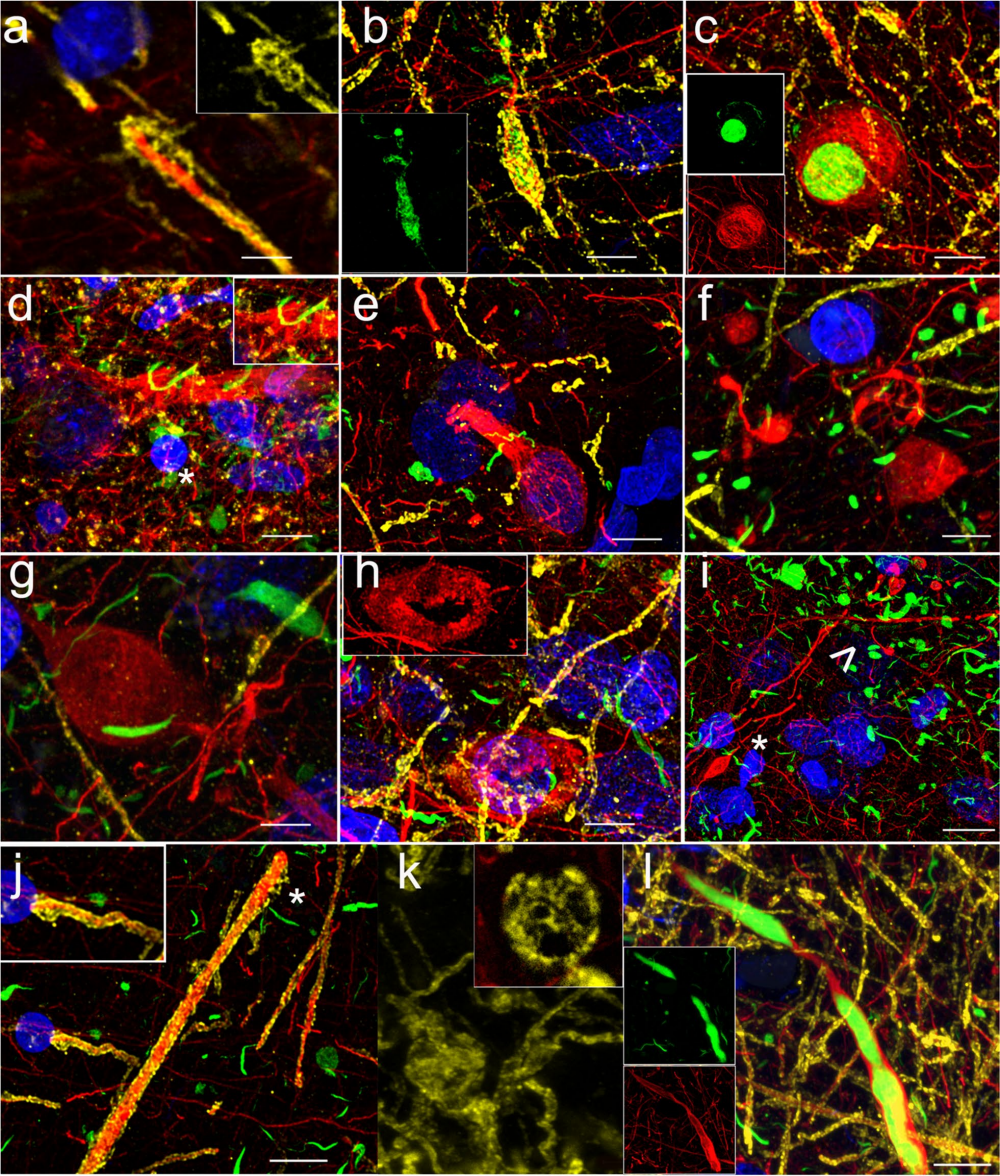
Cytoskeletal (neurofilament heavy-chain; anti-NfH; red, fluorochrome 594) and myelin (anti-PLP; yellow, flourochrome 647) degenerative changes in PD, PDD and DLB donors. In PD_3, myelin blister and detachment from axon (**a**), LN with α-synuclein deposits (green) in myelinated fiber, showing areas of demyelination in the dysgranular insula (**b**), and in the agranular insula a LB (green) surrounded by NfH donut are seen (**c**). NfH fragmentation (**d**, **e**) surrounded by myelin and α-synuclein debris (green) as well as NfH debris and swelling (**f**) were visible in the dysgranular insula in PDD_4. In DLB_2 agranular insula, a large NfH swelling was visible surrounded by α-synuclein deposits (**g**); and NfH donut (**h**), NfH fragmentation, swelling (*) and debris surrounded by α-synuclein aggregates (> , **i**), and focal axonal thickening with myelin blister (**j**) were seen. In DLB_1 myelin swelling was seen with cross-section through the swelling showing multiple vacuoles (**k**). A long LN showing α-synuclein was also present in DLB_2 (**l**). Magnification 1000 × , scale bars: 10 μm. DLB: dementia with Lewy bodies; LB: Lewy body; LN: Lewy neurite; NfH: neurofilament heavy-chain; PD: Parkinson’s disease; PDD: Parkinson’s disease with dementia; PLP: proteolipid protein in myelin

To assess cytoskeletal changes in the anterior insular subregions in PD, PDD and DLB donors, we studied morphological changes in the pattern of NfH and PLP (myelin) immunoreactivity. We observed blister-like myelin formations around thickened axonal segments in both sub-regions, not associated with pSer129 α-synuclein aggregates, in all disease groups (Fig. 4a, j). In DLB, severe myelin changes were observed, including swellings (containing multiple vacuoles), demyelination, and myelin blisters with disrupted axon-myelin continuity (Fig. 4a, j, k). NfH features consisted of fragmentation, extracellular debris and spheroids with single axonal connections, indicating axonal transection (Fig. 4d–g). Extracellular encagement of LBs and donut-shaped NfH aggregates were also observed (Fig. 4c, h). Bulgy LNs were seen in myelinated fibers as well as non-myelinated fibers in the agranular and dysgranular insular sub-regions (Fig. 4b, l).

### Burden of α-synuclein, p-tau, and amyloid-β pathology

**Fig. 5:**
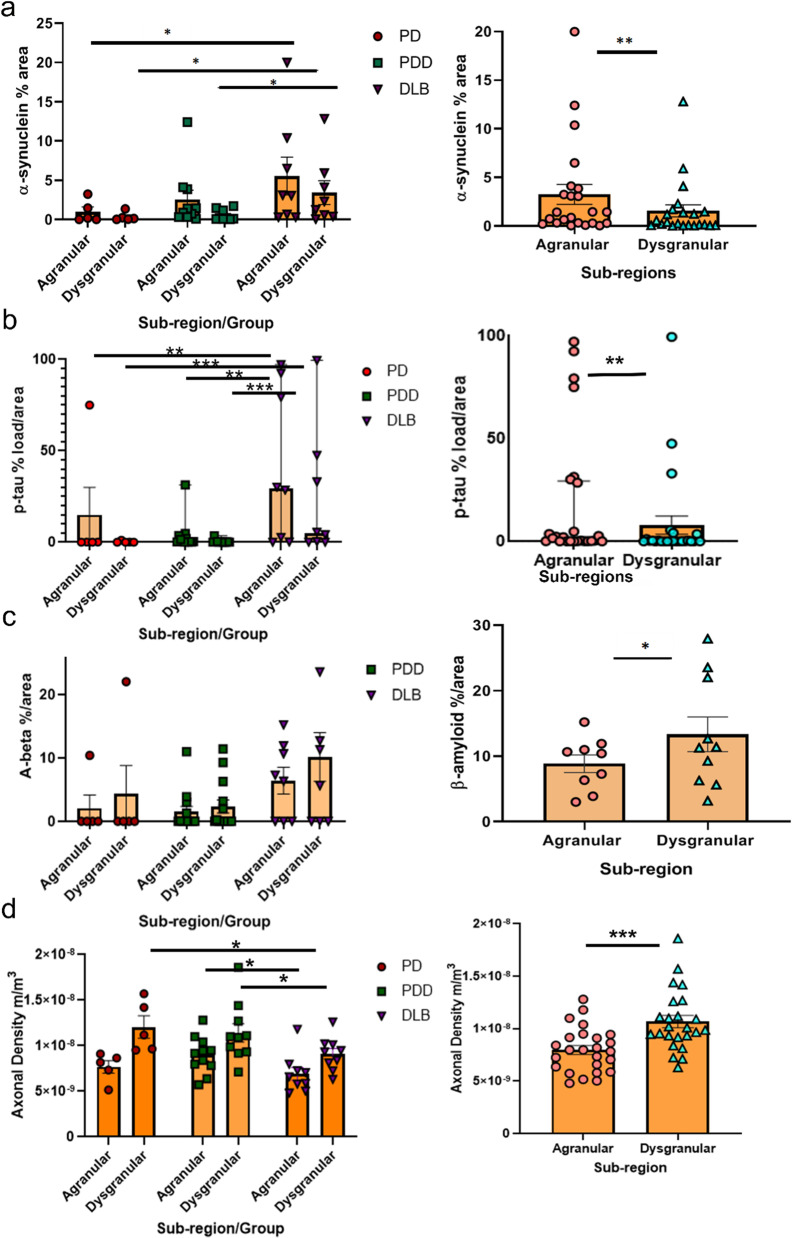
α-Synuclein, p-tau and amyloid-β pathology load in the anterior insular subregions. α-Synuclein pathology load (*n* = 23) was significantly higher in DLB agranular insula (5.7%) compared to PD (1.2%) and PDD (2.3%) and in dysgranular insula (3.4%) compared to PD (0.4%) and PDD (0.6%). Log transformation of scores showed significant differences between DLB and PD in agranular insula (*P* = 0.02) and between DLB and PD as well as PDD in dysgranular insula (*P* = 0.016 and 0.015; respectively); the agranular insula had significantly higher pathology load (3.25%) compared to the dysgranular insula (1.55%) (**a**). p-Tau (*n* = 26) was significantly higher in DLB for both the agranular (41.3%) and dysgranular insula (23.7%) compared to PD (15.1% and 0.25%) and PDD (4% and 0.44%); agranular insula had significantly higher pathology load (18%) compared to the dysgranular insula (7.8%). Amyloid-β (*n* = 26) was not significantly different between groups but the dysgranular insula had significantly higher pathology load (5%) compared to the agranular insula (3%) (**c**). Axonal length density (*n* = 25) based on Bielschowsky silver staining and stereological count showed significantly lower density in the agranular (8 × 10^−9^ m/m^3^) compared to the dysgranular insula ( 1 ×10^-8 ^m/m^3^). The DLB group had significantly lower axonal length density in the agranular insula (6.73 ×10^−9^ m/m^3^) compared to the PDD group (9.17 × 10^−9^ m/m^3^) and in the dysgranular insula compared to both PD and PDD (11.75 ×10^−9^ m/m^3^ and 11.94 × 10^−9^ m/m^3^, respectively) (**d**). Significance: *P < 0.05, **P < 0.01, ***P < 0.001. AD: Alzheimer’s disease; DLB: dementia with Lewy bodies; p-tau: hyperphosphorylated tau; PD: Parkinson’s disease; PDD: Parkinson’s disease dementia

To evaluate whether there were differences in the burden of α-synuclein and co-occurring AD pathology across groups and sub-regions, we quantified the load of protein pathology using area fractionation on free-floating 60-μm thick insular sections. Comparisons between the PD, PDD and DLB groups showed significant differences in the load of α-synuclein (*n* = 23) pathology in the dysgranular insula (*F*(2,18) = 4.9, *P* = 0.019) and a trend for the agranular insula (F(2,18) = 3.5, *P* = 0.054). The DLB group showed significantly higher scores compared to PD and PDD in dysgranular subregion and compared to PD only in agranular subregion (*P* = 0.016 and 0.015 for dysgranular region; *P* = 0.02 for agranular region; Fig. 5). No difference was found between PD and PDD (*P* = 0.86). Between the agranular and dysgranular insula, α-synuclein pathology load was significantly higher in the agranular insula in all groups (mean α-synuclein % area, 3.25% in agranular and 1.55% in dysgranular; *t*(21) = 5.3, *P* < 0.001). For p-tau (*n* = 26), we also observed a significant difference between disease groups for both the agranular (*F*(2,19) = 5.5, *P* = 0.01) and dysgranular insula (*F*(2,19) = 7.1, *P* = 0.005). The DLB group showed a significantly higher load of p-tau compared to PD and PDD groups for both sub-regions (agranular: *P* = 0.01 compared to PD and *P* = 0.006 compared to PDD; dysgranular: *P* = 0.004 compared to both groups). Comparing p-tau pathology within insula sub-regions across groups, the agranular insula showed significantly higher p-tau load compared to the dysgranular insula (mean = 18.0% and 7.8%, respectively; *t*(19) = 5.1, *P* < 0.001). There were no group differences for amyloid-β pathology (*n* = 26; *χ*^2^(2) = 3, *P* = 0.22 for agranular sub-region; *χ*^2^(2) = 2.6, *P* = 0.26 for dysgranular sub-region). However, comparing amyloid-β pathology within the anterior insular sub-regions across groups, the dysgranular insula (mean = 5.0% ± 4.8%) showed significantly higher amyloid-β load compared to the agranular insula (mean = 3.0% ± 8.3%; *t*(25) =  − 2.2, *P* = 0.037) (Fig. 5).

To account for gender differences, analyses were repeated per gender. For α-synuclein load, there were siginificant differences between groups only for females (*F*(2,14) = 49.7, *P* < 0.001). *Post-hoc* analysis showed significant differences between DLB and PD and PDD (*P* < 0.001). Similarly, for p-tau, females showed significant differences between groups (*F*(2,19) = 12.68, *P* < 0.001). The significant differences were found between DLB and PD (*P* = 0.002) as well as PDD (*P* < 0.001). Males also showed a trend of group differences (*F*(2,21) = 3.46, *P* = 0.046), which was due to significant difference between DLB and PDD only (*P* = 0.014).

In summary, across groups, the agranular insular showed more severe α-synuclein and p-tau, but not amyloid-β pathology which was significantly higher in the dysgranular sub-region. Furthermore, α-synuclein pathology in the dysgranular insula significantly differed between DLB and PDD, while p-tau was higher in DLB compared to both PD and PDD. For amyloid-β, no differences between groups were observed.

### Axonal length density is significantly reduced in the agranular insula

Using modified Bielschowsky silver impregnation and stereology, axonal length and axonal length density (*n* = 25) were assessed within the anterior anterior insular sub-regions across all groups. Taking all groups together, the mean total axonal length density for the agranular and dysgranular insula were (8.00 ± 2.14)  ×10^−9^ m/m^3^  and (10.6 ± 2.86) × 10^−9 ^m/m^3^, respectively. The axonal length density was significantly higher in the dysgranular compared to the agranular insula among all groups (*t*(23) =  − 2.7, *P* = 0.01; and *t*(23) =  − 5.7, *P* < 0.001). A significant effect of disease group on axonal length density was only observed in the dysgranular sub-region (*F*(2,23) = 3.7, *P* = 0.04; agranular: F(2,23) = 2.5, *P* = 0.11). Specifically, a significant reduction was found in DLB (8.66 ×10^−9^ m/m^3^) compared to PD (11.75 ×10^−9^ m/m^3^) and PDD donors (11.94 ×10^−9^ m/m^3^) (*P* = 0.04 and 0.02, respectively) (Fig. 5).

### Tau pathology is a major contributor to axonal loss in the anterior insula

Next, we aimed to understand the role of each pathology on axonal degeneration and loss. Using a linear mixed model including all pooled variables (axonal density, disease group, age, p-tau, amyloid-β, and α-synuclein pathology load), p-tau load showed the highest association with axonal length density in both anterior insular subregions (*β* =  − 3.45 × 10^–5^; *F*(1,42) = 4.2; *P* = 0.046). *Post-hoc* analysis showed a significant effect for p-tau on axonal length density in the DLB group (*β* =  − 5.04 × 10^–5^; *t*(24.2) =  − 3.24, *P* = 0.003) compared to PD (*β* =  − 1.5 × 10^–5^; *t*(40.6) =  − 0.42, *P* = 0.67) and PDD (*β* =  − 6.4 × 10^–5^; *t*(41.3) =  − 0.7, *P* = 0.5) as well as for the agranular compared to dysgranular insula (*β* =  − 5 × 10^–5^; *t*(42.3) =  − 3, *P* = 0.004). Testing the effect of other pathology variables on axonal density showed a significant effect for α-synuclein (*β* =  − 3 × 10^–4^; *t*(29) = -3, *P* = 0.006) but no significant effects for amyloid-β (*P* = 0.77). *Post-hoc* analysis for group and sub-region effects on axonal length density showed significant effects for the DLB group (*β* =  − 3.34 × 10^–4^; *t*(45.7) =  − 3.3, *P* = 0.002) compared to PD (*P* = 0.4) and PDD (*P* = 0.7) and agranular compared to dysgranular sub-region (*β* =  − 3 × 10^–4^; *t*(36.6) =  − 2.7, *P* = 0.009) (Fig. 5).

## Discussion

The anterior insular cortex is an important brain region widely connected to the brain, with crucial integrative functions including affective, cognitive, homeostatic, and somatosensory functions [47]. These integrative functions, particularly of the anterior insula, are mainly the result of dense intra/inter axonal insular connections which are disrupted and known to be associated with clinical symptoms of PD [48]. Yet, little is known regarding the underlying neuropathological and degenerative characteristics of insula in PD(D) and DLB, information of which could aid in the identification of primary causes and targets of neurodegeneration. In this study, we aimed to assess axonal degeneration and its relationship with various pathological aggregates in the anterior insular cortex sub-regions in PD(D) and DLB. α-Synuclein and tau pathology load were more severe in the agranular insula of all patient groups, and highest in DLB, while amyloid-β pathology was more severe in the dysgranular insula. In general, the anterior insula invariably harbored a constellation of pathologies including neuronal and astrocytic synucleinopathy, pre-tangles, mature ghost tangles, astrocytic tauopathy, diffuse and dense core amyloid-β plaques, CAA (type I and II) and AGD. p-Tau pathology was found to have the most significant effect on axonal loss, which was most pronounced in the DLB group. Features of axonal degeneration included fragmentation, thinning, thickening, and spheroids as well as cytoskeletal abnormalities including neurofilament degeneration, detachment of the axon-myelin unit and demyelination.

The anterior agranular insula has a distinct allocortical architecture with only four layers, less myelinated fibers as well as late myelination during brain development [49]. Several features may predispose the anterior agranular insula to early degeneration, such as severe impairment of the protein degradation systems and inflammation in response to protein toxicity, leading to build-up of protein aggregates [50]. Moreover, poorly myelinated or unmyelinated fibers are considered more vulnerable to pathology and degeneration, not only in PD but also in other neurodegenerative diseases. The late myelination of the anterior insula during brain development predisposes it to degeneration, as early myelination provides protection to axons through salutatory conduction, reducing the metabolic demands of the neuron and thus oxidative stress [11, 51]. In this study, the anterior agranular insula, compared to the dysgranular sub-region, exhibited more vulnerability to α-synuclein and p-tau pathology, the latter of which had more significant effect on axonal loss. α-Synuclein and p-tau pathology have been shown to co-occur in the same neurons, the two proteins can interact together, and α-synuclein aggregation is thought to induce the phosphorylation of tau, thus promoting tauopathy [52]. This would result in detachment of tau from the cytoskeleton, depolymerization of the microtubule system, tau aggregation and neurofibrillary tangle formation, leading to impaired axonal transport, synaptic loss, and neuronal loss [53].

Amyloid-β pathology, on the other hand, was more prominent in the anterior dysgranular insula, compared to the agranular sub-region. Amyloid-β forms extracellular plaques which mechanically disrupt the function of axons and neurons [54]. Dystrophic neurites found near or within plaques have shown axonal transport deficits contributing to synaptic deficits [55, 56]. Likewise, the early damage of axons and deficient axonal transport would lead to organelle and phagosome accumulation, thereby disrupting protein clearance mechanisms [57, 58]. Accumulating pathology also has an influence on the stability of myelin, leading to further loss of axonal maintenance and reduced neurofilament transport [59]. Of note, recent evidence shows that approximately 40% of amyloid-β is produced in axons [60, 61]. Put together, this is particularly relevant in DLB where the co-occurrence of all three pathologies as well as CAA has commonly been reported [62]. The presence of these concomitant pathologies is associated with more severe neurodegeneration and rapid disease course, leading towards cognitive decline and dementia, to which pathology in the anterior insula would largely contribute [62–64]. Previous studies have shown that AD pathology, namely NFTs and amyloid-β oligomers, can inherently contribute to axonal degeneration through microtubule destabilization, decreased mitochondrial density and energy depletion, resulting in oxidative stress, impaired calcium homeostasis and axonal transport [65]. Furthermore, similar to the synergistic effect of both tau and α-synuclein, an interplay between amyloid-β and tau promotes their transformation into toxic states, further inducing axonal degeneration [66, 67]. Besides AD pathology, AGD, a form of tauopathy, was also found in the anterior insula in DLB. AGD has a typical anterior-to-posterior distribution gradient and is linked to cognitive deficits, thus adding to the cluster of protein aggregates present in this disease group [68].

In this study, axonal loss, studied as axonal length density, was found most pronounced in the agranular insula. However, axonal loss in the dysgranular insula was most severe in the DLB group compared to both PDD and PD and significantly correlated with cognitive impairment. These results support our previous study showing that cognitive impairment in PD is related to dysgranular insular functional connectivity [48]. Moreover, recent MRI studies highlight the importance of anterior insular degeneration in DLB, indicating that the anterior dorsal (dysgranular) insula shows early atrophy in DLB, and could function as a potential biomarker [69] allowing differentiation between PDD and DLB.

**Fig. 6:**
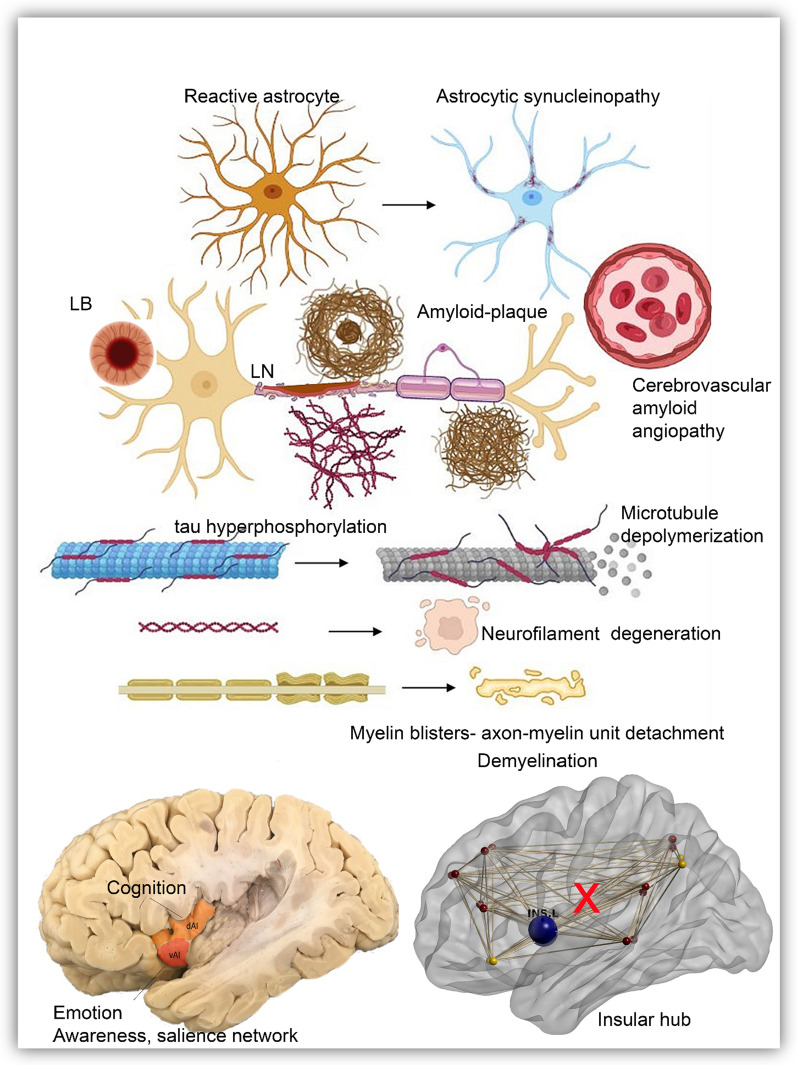
Schematic drawing of proteinopathy and axonal degeneration in the anterior insula in PD, PDD and DLB. The neuron, its axon, axonal cytosketon, myelin, and supportive cells such as astrocytes are all involved in the degenerative process in PD, PDD and DLB with more severe features in DLB. Astrocytes are reactive and take up α-synuclein deposits ending in their degeneration. The axon is affected at multiple levels; it contains α-synuclein deposits (LBs and LNs) which impede axonal transport. Neurofibrillary tangles (NFT) contain hyperphosphorylated tau (p-tau) that begins as pre-tangles and matures into ghost tangles. The hyperphosphorylation and accumulation of p-tau impairs the axonal cytoskeleton and leads to depolymerization of the microtubule. p-Tau and α-synuclein can also stimulate each other’s aggregation, speeding up degeneration. Amyloid-β plaques, which are more severe in the dysgranular insula, contribute to impaired cellular trafficking and myelin instability. Amyloid-β deposits in and surrounding blood vessels, CAA, lead to poor clearance of abnormal proteins and blood–brain barrier dysfunction. Finally, myelinated axons are also affected through axon-myelin unit detachment and demyelination, which affects conduction velocity and axonal support. All together, axonal length, integrity and cellular trafficking become impaired in the anterior insular subregions in PD, PDD and most severe in DLB donors with consequent impairment of insular functions as a brain hub and emotional/cognitive deficits as a result. CAA: cerebrovascular amyloid angiopathy; DLB: Dementia with Lewy bodies; LB: Lewy body; LN: Lewy neurite; PD: Parkinson’s disease; p-tau: hyperphosphorylated tau

Using Bielschowsky silver impregnation, we observed various degenerative axonal features, including thickening, thinning, spheroids, and bulbuous swellings in the vicinity of β-amyloid plaques, many of which were also observed with neurofilament-heavy chain (NfH) immunostaining (as summarized in Fig. 6). Moreover, we found NfH spheroids fully encapsulating LBs and axonal spheroids with single axonal connections, indicating axonal transection. Neurofilaments are important cytoskeletal components regulating axonal caliber and growth as well as allowing docking and transport of organelles. Hyperphosphorylation of neurofilament-light chain (NfL) is thought to cause aggregation, impaired docking of motor proteins as well as impaired transport [70]. Recently, it has been found that NfL is present within LBs forming a shell-like structure along with other cytoskeletal structures encapsulating accumulated materials within LBs, such as lipids and proteins [71, 72]. Meanwhile, serum NfL biomarker levels are thought to reflect axonal neurodegeneration and correlate with cognitive decline in PD and AD [73–75]. Here, we show that NfH is also severely affected in the anterior insula. NfH subunits generally increase within axons as they mature, and play an important role in axonal support by allowing axonal stability which is important in providing docking sites for interactions with motor proteins and thus providing neurons, particularly those with long axons, the opportunity to conserve energy. It is also known that abnormal NfH phosphorylation generally leads to slower axonal transport, thus affecting disease pathogenesis [76].

Likewise, the myelin sheath is an important axonal component providing support through glial axonal maintenance, allowing transportation of neurofilament and phosphorylation, leading to higher axonal caliber, and faster transmission through the nodes of ranvier [59]. In this study, we reported myelin blisters, detachment from the axon-myelin unit, with thickening and bleb-like formation of the underlying neurofilament, as well as presence of LNs in myelinated nerve fibers showing patches of demyelination (as summarized in Fig. 6). Myelin blisters have been documented in multiple sclerosis where they cause disruption of the axon-myelin unit, triggering abnormal calcium signalling and myelin degeneration [77]. Generally, myelination within the insula shows a decreasing gradient from posterior to anterior with late and scarce myelination of the agranular anterior insula [78]. To our knowledge, myelin changes have not been well-described in PD and DLB, yet appear to undergo early degeneration thus warranting further analysis. We previously showed swollen axons and myelin deficits with cryogenic X-ray nanotomography and illustrated ultrastructural myelin deficits in the substantia nigra in PD [79]. In the current study, we show that similar axonal deficits are present in the anterior insula in PD(D) and DLB.

This study, although the first to elucidate features of axonal degeneration and pathology in the anterior insula in PD(D) and DLB, has several limitations. First, we have a limited sample size, particularly for the PD group. Most PD cases at the time of autopsy had records of severe cognitive decline and/or dementia (PDD). As such, brain tissues of PD donors without dementia are scarce. Yet future studies with larger cohorts at different disease stages could shed more light on the progression of axonal degeneration across PD and DLB. Second, our study lacks controls. Although 13 controls were carefully selected and processed, the majority did not fulfill the quality requirements for the Bielschowsky staining and were therefore excluded. This is likely the result of postmortem changes and tissue handling processes non-compliant with Bielschowsky impregnation specifications. Third, we have explored the role of astrocytic reactivity and relationship with α-synuclein (syn-1) in PD(D) and DLB brains; however, further analysis of other neuroinflammatory markers in relation to various protein aggregates within the insula would provide more comprehensive knowledge on the precise role of inflammation in the insula and its contribution to disease progression. Furthermore, future studies providing quantitative results on cytoskeletal changes, including myelin and neurofilament changes, in diseased patient groups compared to healthy controls would greatly advance our understanding of the extent of axonal damage and potentially lead to identification of novel therapeutic markers.

## Conclusions

Our study provides evidence that in PDD and DLB, the anterior insula is highly selectively vulnerable to pathological aggregates. It harbors a constellation of pathological protein deposits including α-synuclein aggregates, NFT (p-tau), amyloid-β plaques, CAA and AGD, which are most severe in DLB. Axonal loss is significantly more severe in DLB and degenerative axonal features include axonal swelling and transection along with cytoskeletal neurofilament and myelin abnormalities in the anterior insula of all disease groups. While the agranular insula is severely affected with p-tau and α-synuclein aggregates, amyloid-β pathology shows preferential distribution in the dysgranular insula. These abnormalities are most severe in the DLB patient group, providing neuropathological evidence of anterior insular damage in this disease. Given the importance of the anterior insula as a hub connecting various brain regions and integrating emotional, autonomic, and cognitive functions, the insula provides a unique opportunity to understand cyto-architecture-specific vulnerability to pathology, degeneration, and widespread functional deficits.

## Data Availability

The datasets during and/or analysed during the current study are available from the corresponding author on reasonable request.

## Abbreviations

AGD: Argyrophillic grain disease
ARTAG: Age-related tauopathy of the astroglia
CAA: Cerebral amyloid angiopathy
CE: Coefficient of error
CLSM: Confocal laser-scanning microscopy
DLB: Dementia with Lewy bodies
GFAP: Glial fibrillary acid protein
H&Y: Hoehn and Yahr
NBB: Netherlands brain bank
NfH: Neurofilament heavy-chain
NfL: Neurofilament light-chain
NFT: Neurofibrillary tangles
PD: Parkinson’s disease
PDD: Parkinson’s disease dementia
PLP: Proteolipid protein
p-tau: Phosphorylated tau
ROI: Region of interest
